# Aqueous Gold Nanoparticles Generated by AC and Pulse-Power-Driven Plasma Jet

**DOI:** 10.3390/nano9101488

**Published:** 2019-10-18

**Authors:** Pengcheng Xie, Yi Qi, Ruixue Wang, Jina Wu, Xiaosen Li

**Affiliations:** 1 College of Mechanical and Electrical Engineering, Beijing University of Chemical Technology, Beijing 100029, China; xiepc@mail.buct.edu.cn (P.X.); 2018210368@mail.buct.edu.cn (Y.Q.); 2 State Key Laboratory of Organic-Inorganic Composites, Beijing University of Chemical Technology, Beijing 100029, China; 3 State Key Laboratory of NBC Protection for Civilian, Beijing 102205, China; wujina09@163.com

**Keywords:** aqueous gold nanoparticles, AC-powered plasma jet, pulse-powered plasma jet, gold nanoparticle generation, chemical reactions

## Abstract

In this study, we developed a simple-to-use approach based on an atmospheric pressure plasma jet to synthesize aqueous Au nanoparticles (AuNP). Special attention was paid to the different reaction dynamics and AuNP properties under AC and pulse-power-driven plasma jets (A-Jet and P-Jet, respectively). The morphology of the AuNP, optical emissions, and chemical reactions were analyzed. Further, a copper mesh was placed above the reaction cell to evaluate the role of electrons and neutral species reduction. A visible color change was observed after the A-Jet treatment for 30 s, while it took 3 min for the P-Jet. The A-Jet treatment presented a much higher AuNP growth rate and a smaller AuNP diameter compared with the P-Jet treatment. Further analysis revealed an increase in chemical concentrations (Cl^−^ and H_2_O_2_) and liquid conductivity after plasma treatment, with a higher increased amplitude for the A-Jet case. Moreover, the electrons alone had little effect on AuNP generation, while neutral species showed a clear Au^+^ reduction effect, and a unique coupling effect between both reactions was observed. The different reaction dynamics between the A-Jet and P-Jet were attributed to their different local heating effects and different discharge power during the reaction.

## 1. Introduction

High-quality gold nanoparticles (AuNPs) play a crucial role in numerous fields, including catalysis, optoelectronics, medical imaging, and sensors [1,2]. Highly purified surfactant-free AuNPs are very important because residual components limit their applications. Numerous methods, including chemical reduction, biosynthesis, and laser ablation, have been reported for AuNP synthesis [3,4]. However, the high cost associated with the synthesis of monodisperse size and shape-controlled nanoparticles and the use of toxic reducing agents makes them undesirable [5].

Plasma–liquid interaction has received much attention as a novel nanoparticle generation approach [6]. The electron energy generated by atmospheric pressure plasma can reach up to a few electron volts; thus, it is able to initiate the direct reduction of metal ions in the gas–liquid interface. Other oxidative species, such as OH∙ and H_2_O_2_, can have possible reactions with chloroauric acid [AuCl*_x_*(OH)*_4−x_*]^−^ to form AuNP because AuCl^−^_x_ has a strong oxidation ability [7]. Various types of plasma can be applied to produce nanoparticles [8]: (1) remote plasma or plasma jet: gas discharge between an electrode and the electrolyte surface, (2) direct plasma: discharge between the electrodes. Microplasma, as one of the remote plasmas, usually works as a cathode electrode [9]. The high-energy electrons are believed to be responsible for initiating AuNP growth [10]. Normally, the electrolytic cell is driven by a DC power supply with several kV, and a large resistor (100 kΩ) is connected to the circuit to limit the discharge current. Most of the power is consumed on the resistor instead of discharge. In addition, due to the small diameter of microplasma (hundreds of micrometers), the production rate is limited. In direct plasma, two metallic electrodes were immersed in liquids [11]. By applying radio frequency (RF) power, metallic nanoparticles are produced through the erosion of a metallic electrode exposed to plasma. The particle size is determined by the quenching rate of the surrounding water.

Recently, the continuous synthesis of colloidal gold nanoparticles by introducing liquid droplets into a plasma reactor has been proposed [12,13]. The picoliter reactor volume and droplet effect result in a high synthesis rate. However, the ability to scale-up the reactor might make this technology more practical. Ionic liquids have a very low vapor pressure and thus can operate at a low pressure where traditional large-sized processing plasmas can be applied [14]. Although there is no need for additional surfactant to stabilize the synthesized NPs, it is difficult to change their surface function. Most importantly, specific metal salts or ionic liquids are required due to the low solubility of many metal salts in ionic salts [15].

Atmospheric pressure plasma, working at room temperature, has drawn an immense amount of interest due to it being easy to scale up, its low cost, and its compact size [16,17,18]. A plasma jet, generated in open air rather than in confined chambers, can be used for direct treatment without the limitation of the object’s size [19]. However, the treatment area of single plasma is small (less than 1 cm^2^) due to its inherent structure [20]. One convenient method is to form a jet array by grouping a number of individual plasma jets units together. Although the interaction mechanisms between individual jets require further investigation, a 2D jet array with 45 single jets has been successfully implemented to form a large treatment area [21]. The scale-up ability of plasma jets makes them practical for further industrial application.

In this study, we developed a one-step approach based on an atmospheric pressure plasma jet that directly interacts with liquids to generate aqueous AuNPs. By applying the plasma jet above the liquid surface, the AuNP can be produced by plasma–liquid interaction. Different from the microplasma mentioned above, there is no secondary electrode needed. In addition, the treatment area of the plasma jet can be enlarged by grouping multiply plasma jets together, which provides the possibility for industrial application. The different reaction dynamics and AuNP properties were compared with AC and pulse-power-driven plasma jets (A-Jet and P-Jet, respectively). The plasma-induced chemistry was studied by monitoring different chemical concentrations (Cl^−^, H_2_O_2_, and NO_3_), the pH value, and the conductivity of the liquid during reaction processes. The role of electrons and neutral species for Au^+^ reduction was analyzed by isolating these two factors into different reaction cells. Finally, the chemical reactions in the liquid by A-Jet and P-Jet were analyzed to explain the synthesis mechanisms involved in the A-Jet and the P-Jet.

## 2. Materials and Methods

In order to synthesize aqueous AuNPs, an atmospheric pressure plasma jet was placed 1 cm above an aqueous solution mixed with HAuCl_4_ and sodium citrate (total volume: 4 mL). The plasma jet was constructed with a typical needle–ring electrode structure, the details of which can be found in reference [22]. A hollow stainless-steel tube was inserted into a quartz tube (inner diameter: 1.5 mm, wall thickness: 0.1 mm, length: 150 mm), working as a high-voltage electrode. Copper tape with a width of 10 mm and a thickness of 180 μm was wrapped around the quartz tube surface and worked as a ground electrode. HAuCl_4_ and sodium citrate were purchased from Sinopharm and were used without further purification. Once the plasma jet was ignited, high-energy electrons, reactive species, and UV were generated in the gas phase. These species subsequently went through the plasma–liquid interface to the bulk liquid. In the liquid region, the reduction of Au^+^ into Au^0^ occurred, and AuNPs started to grow with the existence of the stabilizer (sodium citrate). Due to the high recombination rate of reactive species, only long-lived species (such as H_2_O_2_, OH-, NO^−^_2_, NO^−^_3_) penetrated into the liquid. At the same time, the color of the HAuCl_4_ solution turned gradually from light yellow to pink, which indicated the formation of AuNP. After several seconds or minutes (depending on power supply) of the plasma jet treatment, the solution containing AuNP was collected.

**Figure 1:**
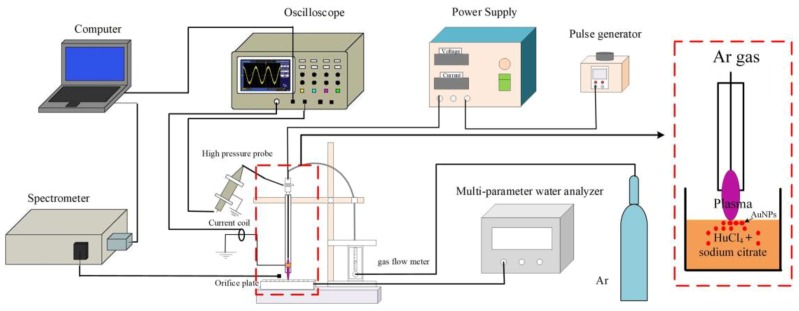
A schematic diagram of the plasma jet setup used for AuNP synthesis (inset shows enlarged diagram of plasma jet–liquid system).

The plasma jet was powered either by an AC power supply (Nanjing Suman Plasma Technology, CTP-2000K, Nanjing, China) or a pulsed power supply (Xi’an Smart Maple Electronic Technology, HVP-22P, Xi’an, China), and the discharge parameters were verified. Research-grade Ar gas (99.999%) with a gas flow rate of 2 slm (standard liter per minute) was used as the working gas. Once the plasma was ignited, the high-energy electrons that cascaded into the liquid solution were responsible for the initiation of AuNP growth. The schematic diagram of the plasma AuNP synthesis system is shown in Figure 1.

The voltage and current characteristics were monitored by a high-voltage probe (Tektronix, P6015A, 1000:1, OR, USA) and a current probe (Pearson, model4100, 1 V/A) through a digital oscilloscope (Lecory WR204Xi, NYC, USA), respectively. The input power was calculated by the voltage–charge Lissajous method. A small capacitor with a capacitance of 1000 μF was connected to the plasma jet ground electrode. The area (*A*) enclosed by the power voltage, and the capacitor charge (Q) was calculated. The input power (*P*) was determined by

(1) P=fA,

where f is the pulse frequency.

A fiber optic cable was placed near the exit nozzle of the plasma jet to guide the light emission to the spectrometer (Ocean optics, Maya 2000 pro, FL, USA). Visible images of the as-synthesized AuNPs were captured by a digital SLR camera (Nikon D3200, Tkyo, Japan) coupled with a zoom lens (Nikkor, S-line, Nikon, Tkyo, Japan) with an exposure time of 0.5 ms. The AuNPs were characterized by an ultraviolet–visible absorption spectrometer (IMPLEN Nanophotometr N60, MUC, Germany) in the wavelength range from 200 to 900 nm. The size and shape distribution of the AuNPs was analyzed by a transmission electron microscope (TEM, TECNAIF30, OR, USA). A selected area electron diffraction (SAED) device coupled with the TEM was used to identify the crystal structure of the AuNPs.

The concentration of Cl^−^ was measured by a water quality analyzer (Leici DZS-708, Shanghai, China). For Cl^−^ measurement, the measuring electrode and reference electrode were immersed in a 0.001 mol/L KCl standard solution for 2 h. KCl solutions with five gradients (100, 10, 1, 0.1, and 0.01 mmol/L) were used for Cl^−^ calibration. After each measurement, the electrodes were rinsed with deionized water to reduce the experimental error. The conductivity and pH value of the samples were measured by a conductivity meter (Leici TM-03 Pen-shaped conductivity meter, Shanghai China) and a pH meter (Leici PHSJ-3F, Shanghai, China), respectively. A hydrogen peroxide kit (LOHAND Test Strips Series 0–25 mg/L, Hangzhou, China) measured the concentration of hydrogen peroxide.

## 3. Results

### 3.1. Characterization of A-Jet and P-Jet

**Figure 2:**
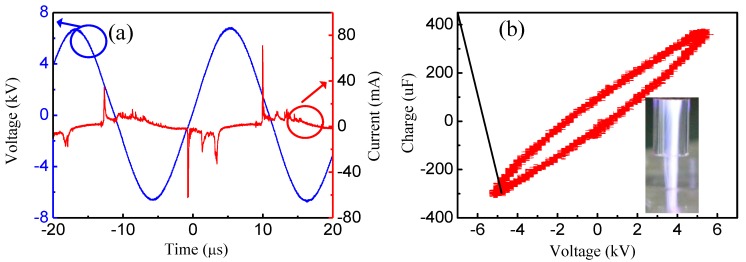
(**a**) Voltage and current discharge waveforms and (**b**) the corresponding Lissajous figure (The inset shows discharge image of A-Jet).

Figure 2 represents the typical characteristics of A-Jet by measuring the voltage-current waveform, the calculated Lissajous figure, and the discharge image. During discharge, the applied voltage and frequency were kept consistently as 6.8 kV and 90 kHz, respectively. The discharge occurred at both voltage rise and fall times during one discharge period, as shown in Figure 2a. There were several small current peaks followed by the main current peak at pulse rise and fall times, which confirmed a filamentary discharge mode in our condition (inset image). The development of a filamentary micro-discharge can be divided into three stages [23]: (1) pre-breakdown stage with negative charge accumulation, (2) ionization wave propagation towards the cathode, and (3) discharge filament bridged the gap, and a bright plasma channel is formed. To calculate the discharge power during discharge, a 100 μF capacitor was series-connected in the circuit. The obtained Lissajous figure is shown in Figure 2b. The average power is equal to the product of the repetition frequency and the energy in a discharge period. So, the discharge power was calculated as 30.1 W. The input power of the AC power supply was 68.1 W; thus, the power efficiency was 45%.

**Figure 3:**
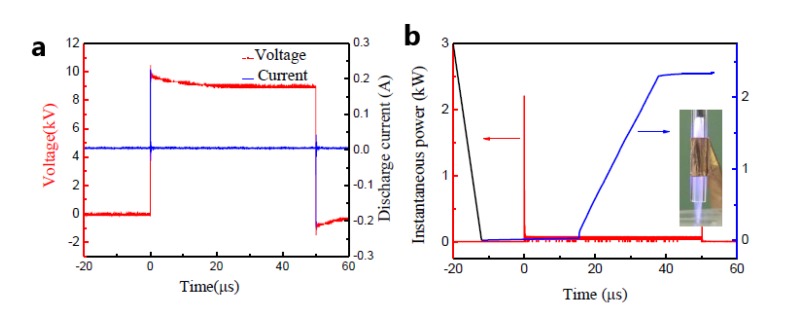
(**a**) Voltage and current discharge waveforms and (**b**) instantaneous power and energy waveforms of the P-Jet (the inset shows the discharge image of P-Jet).

Different from the AC power supply, pulsed power supply with rapid pulse rise time provides high reduced electric field intensity (E/N) to accelerate electrons. In a pulsed discharge mode, high energy electrons are believed to ionize the gas to generate secondary electrons instead of depending on space ionization [24], so, the discharge is more spatially uniform, and the gas temperature is much lower in P-Jet compared to A-Jet. Figure 3 shows the typical waveforms of applied voltage, plasma current, discharge image, and consumed energy during one pulse duration. The discharge power was set as follows: a pulse rise and fall time of 50 ns, a pulse duration time of 5 μs, and a pulse frequency of 8 kHz. One can see that the amplitudes of applied voltage and plasma current of the pulsed plasma jet were 8.5 kV and 0.2 A, respectively (Figure 3a), with bipolar plasma current behavior. The instantaneous power consumption was 2.2 kW, and the energy consumption during one pulse duration was 2.34 mJ, respectively (Figure 3b). With an applied frequency of 8 kHz in this case, the energy power consumption was 16.4 W, which was much lower than that of the A-Jet (30.1 W). Compared to A-Jet, the discharge of P-Jet was much gentler and more homogenous, as shown in the inset image.

### 3.2. Synthesis of AuNP by A-Jet and P-Jet

**Figure 4:**
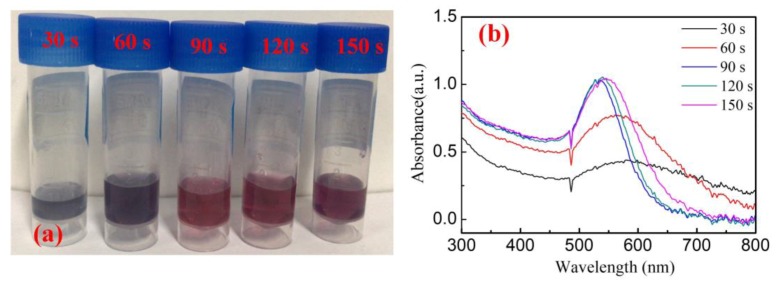
(**a**) Photos and (**b**) UV–Vis absorption spectra of AuNP for process times of 30, 60, 90, 120, and 150 s.

To generate AuNP, 1 mL HAuCl_4_ (1.214 mM) and 3 mL sodium citrate (34 mM) solutions were mixed together and treated with the A-Jet for different times. The time dependence of AuNP generation was studied first. As shown in Figure 4a, with a plasma jet treatment of 30 s, the mixture solution changed from a shallow yellow to a dark violet, confirming the generation of AuNPs. As the plasma treatment time increased, the solution gradually became a red color. The shift in color was basically due to light absorption, depending on the particle size. When the size of the nanoparticles decreased, smaller wavelengths would be absorbed, and, so, a red color would be reflected. The absorption peak showed a similar trend: the absorption peak shifted towards lower wavelengths as the process time increased (Figure 4b). The absorption peaks were centered at 584, 566, and 535 nm for different plasma processing times of 30, 60, and 90 s, respectively. As the plasma treatment time increased to 120 s, the absorption peak intensity stopped increasing, and the central absorption peak shifted towards higher wavelengths. This phenomenon was more obvious when the plasma processing time increased to 150 s, at which time the central absorption peak increased to 545 nm, and the peak width became wider. This indicated that after 90 s, the Au^+^ in the solution was totally consumed and AuNP began to aggregate. After 90 s of reaction, the synthesized AuNPs were isolated from the solution by centrifugation for 20 min at 1 × 10^4^ rad/min and dried overnight at room temperature. A total of 9 mg of AuNP was collected, and the formation rate was calculated as 0.4 mg/s.

**Figure 5:**
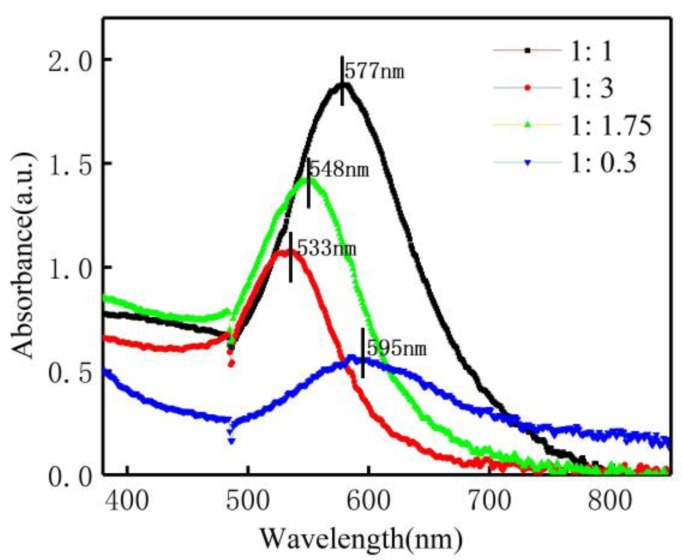
UV–Vis absorption spectra of AuNP for different HAuCl_4_/sodium citrate ratios: (**a**) 1:1, (**b**) 1:3, (**c**) 1:1.75, and (**d**) 1:0.3. The total volume was kept at 40 mL, and the plasma treatment time was 90 s.

**Figure 6:**
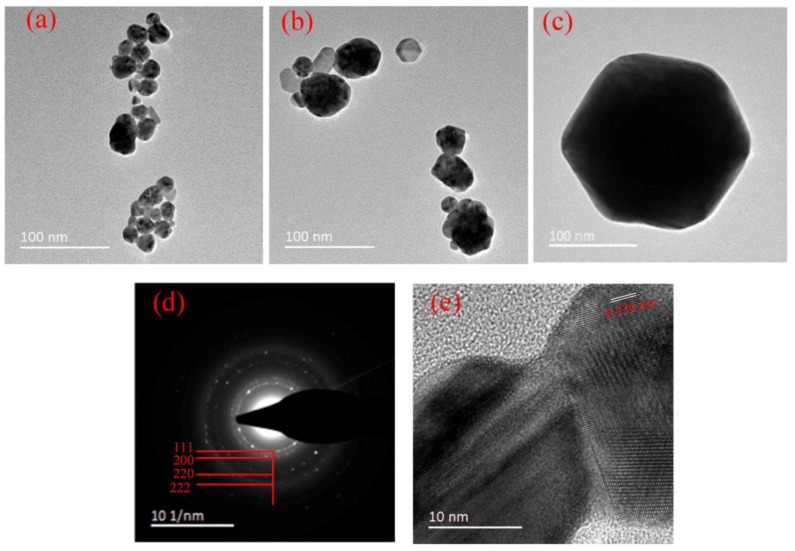
TEM images with different HAuCl_4_/sodium citrate ratios: (**a**) 1:3, (**b**) 1:1.75, and (**c**) 1:0.3. (**d**) Selected area electron diffraction (SAED) pattern of an ensemble of AuNP, and (**e**) image of a typical single crystal of AuNP.

With variations in the HAuCl_4_/sodium citrate ratio, the absorption peaks of AuNP also changed (Figure 5). The central absorption peak position shifted to lower wavelengths with decreasing of the HAuCl_4_/sodium citrate ratio. This “blue shift” of the peak indicated a decrease in the average nanoparticle size as the sodium citrate volume increased. This was understandable since the sodium citrate acted as a stabilizer as well as a reducing agent. In addition, the absorption peak intensity decreased as the sodium citrate volume increased due to fewer Au seeds provided by HAuCl_4_. A higher volume of sodium citrate led to more Au seeds and prevented the aggregation of AuNPs [25]. This was further confirmed by a broad distribution of UV–Vis absorption spectra with a very small volume of sodium citrate. The TEM images (Figure 6a–c) showed that the AuNP were generally of a range of shapes (spherical, cylindrical, and hexagon). The average diameters of the synthesized AuNPs were 18.2 ± 9.0, 32.9 ± 14.1, and 180.6 ± 20.5 nm with HAuCl_4_/sodium citrate ratios of 1:3, 1:1.75, and 1:0.3 (Figure 6a–c), respectively. The size distribution of the synthesized AuNPs measured by TEM was consistent with the UV-Vis spectra results (Figure 5), which confirmed that the higher sodium citrate concentration reduced the AuNP diameters. The dotted rings in the SAED pattern in Figure 6d suggest that these AuNP had a pronounced crystal structure. The *d*-spacing of the rings suggests that the dotted rings represented the Bragg reflection of the [111], [200], [220], and [222] crustal planes, indicating a face-centered cubic (fcc) crystal structure. In addition, the high-resolution TEM (HRTEM) image of one typical single AuNP (Figure 5e) showed a crystal lattice fringe spacing of 0.236 nm, corresponding to the [111] lattice planes of the AuNP.

**Figure 7:**
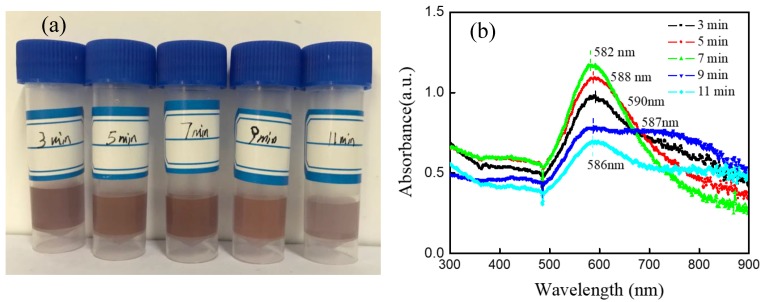
(**a**) Photos and (**b**) UV–Vis absorption spectra of AuNP for process times of 3, 5, 7, 9, and 11 min under P-Jet treatment (pulse frequency: 8 kHz, voltage: 8 kV, HAuCl_4_/sodium citrate ratio of 1:3).

Unlike the A-Jet case, the mixture solution did not change colors until 3 min of pulse plasma treatment (HAuCl_4_/sodium citrate ratio of 1:3). Further, the solution presented a brick-red color after 3 min of reaction and seemed cloudy (Figure 7a). Accordingly, the UV–Vis absorption peak was centered at 590 nm, which confirmed a large AuNP diameter (Figure 7b). As the pulsed plasma treatment time increased, the UV–Vis spectra shifted to a lower wavelength (582 nm at 7 min). The further increase of reaction time resulted in decreased absorption peak intensity and a broad absorption peak width (indicating a broad size distribution). After 7 min, the Au^+^ in the solution was totally consumed and AuNP began to aggregate. The generated AuNP were collected after 7 min reaction, and the formation rate was calculated as 9.5 μg/s. Comparing the reactions initiated by the A-Jet and the P-Jet, the following differences were found: (1) the AuNP generation rate by the A-Jet was much faster than that of the P-Jet; (2) the AuNP size distribution was narrower in the A-Jet case; and (3) the AuNP size control range was broader in the A-Jet case. These differences were due to the different power consumption rates and different chemical reaction pathways introduced by the A-Jet and the P-Jet, which is well explained in the next section.

**Figure 8:**
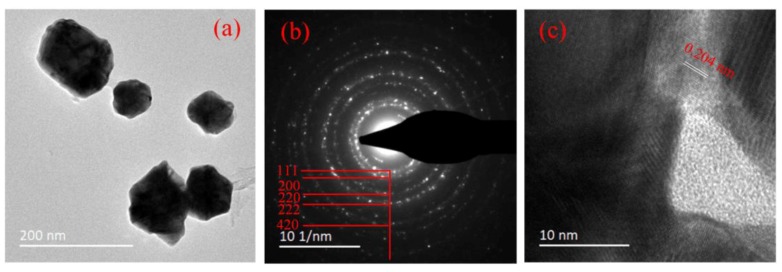
(**a**) TEM images of AuNP produced with a HAuCl_4_/sodium citrate ratio of 1:3 with pulsed plasma treatment for 7 min. (**b**) SAED pattern of an ensemble of AuNP. (**c**) high-resolution TEM (HRTEM) image of a typical single crystal of AuNP.

The TEM images of AuNP produced with a HAuCl_4_/sodium citrate ratio of 1:3 for 7 min are shown in Figure 8. As shown in Figure 8a, the AuNP also presented a broad range of shapes, including spheres and polygons, and had an average diameter of 82.5 ± 21.5 nm. The dotted rings in the SAED pattern in Figure 8b represent the Bragg reflection of the [111], [200], [220], [222], and [420] crystal planes, indicating an fcc crystal structure. The HRTEM image of one typical single AuNP (Figure 8c) showed a crystal lattice fringe spacing of 0.204 nm (corresponding to the [200] lattice planes), which confirmed the crystallinity of the particles.

**Figure 9:**
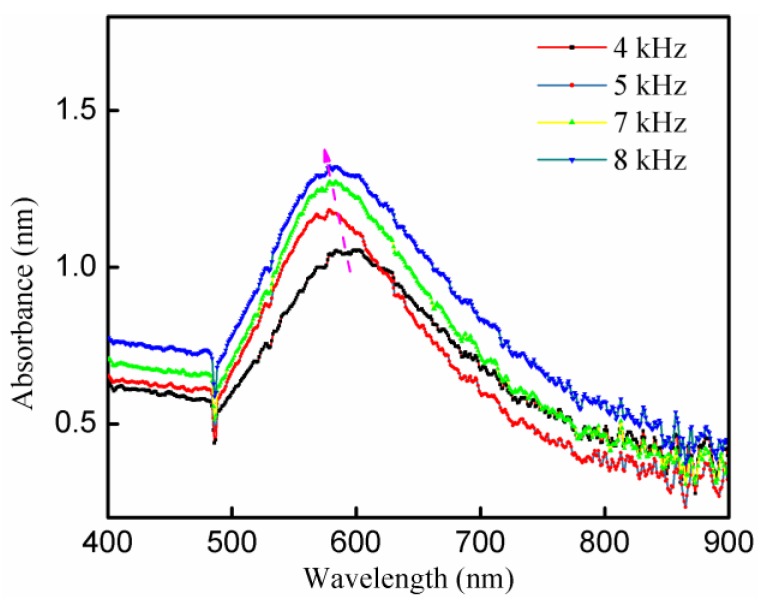
UV–Vis absorption spectra of AuNP under different pulse frequencies (applied voltage: 8.4 kV, treatment time: 7 min, HAuCl_4_/sodium citrate ratio of 1:3).

As the diameter of synthesized AuNP increased with the decrease of sodium citrate concentration, and with HAuCl_4_/sodium citrate ratio of 1:3, the average diameter of particle size was 82.5 ± 21.5 nm. So, there was no need to change the HAuCl_4_/sodium citrate ratio as we did in A-Jet. Instead, the pulse repetition frequency was varied. Figure 9 shows that the intensity of the UV–Vis absorption peaks varied with the changes in pulse frequency. With the increase of pulse frequency, the UV–Vis absorption peak presented a blue shift, and the peak intensity increased. The increase of pulse frequency enhanced the input energy, thus corresponding to a higher AuNP generation rate.

Table 1 and Table 2 summarized the influence of variation of experimental parameters on AuNP properties by A-Jet and P-Jet, respectively. The particle sizes and shapes were obtained by TEM images of the synthesized AuNP. For the particle size distribution, around 100 particles were measured, and their average particle size was calculated. The AuNP with spherical, cylindrical, and hexagon shapes were found in all cases, confirming that these parameters had a little influence on particle shape. In other words, it was very difficult to control particle shape in our system. However, the particle diameters changed. For time-dependence, A-Jet and P-Jet showed a similar tendency. The UV–Vis absorption peak first decreased then increased with a plasma treatment time increase. In the first stage, a loose self-assembly of small Au nanoclusters formed coarse particles and disassembled into small nanoparticles at a low pH value. With the decrease of the AuHCl_4_/sodium citrate ratio, the UV-Vis absorption peak showed “red shift”, and particle size increased in A-Jet. It was interesting to see that the diameter of AuNP in A-Jet and P-Jet was 18.2 ± 9.0 nm and 82.5 ± 21.5 nm at AuHCl_4_/sodium citrate ratio of 1:3, indicating smaller AuNP were produced with A-Jet. A further decrease in sodium citrate ratio concentration would induce a larger particle size. So, it was hard to generate AuNP with a smaller size with P-Jet. That was why the variation of the AuHCl_4_/sodium citrate ratio was not conducted in the P-Jet case. Instead, we changed the pulse repetition frequency and found that although the UV-Vis spectra peak moved to a lower wavelength, the particle diameter did not change too much if we compared it to the A-Jet case.

### 3.3. Plasma-Induced Chemistry Involved in A-Jet and P-Jet

**Figure 10:**
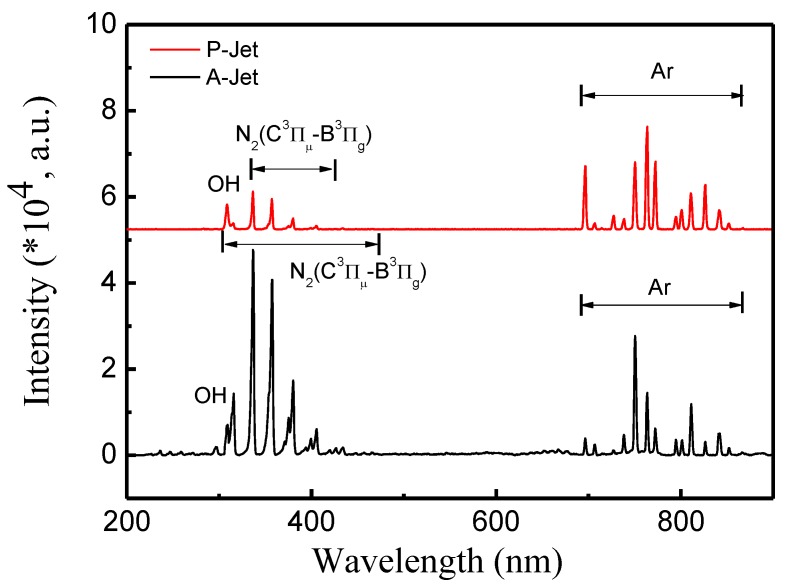
Optical emission spectra of the A-Jet and P-Jet near the liquid surface.

To explain the mechanism involved in A-Jet and P-Jet AuNP synthesis, optical emission spectroscopy was used to investigate the active species generated in the plasma jet. As shown in Figure 10, OH∙ emission at 306–309 nm and N_2_ second positive system (*C^3^Π_μ_-B^3^Π_g_*) at 337, 353, 380, and 405 nm were clearly observable in both cases; however, the peak intensity in the A-Jet case was much higher than in the P-Jet case. Similar Ar emissions from 656 to 850 nm presented in both cases and the intensity of these emissions were close. Near the gas–liquid interface, the dissociative electron attachment of H_2_O formed OH∙ radicals and subsequently combined into long-lived species (H_2_O_2_), which played an important role in the AuNPs synthesis:

(2) $$e_{\mathrm{gas}}^{-}+H_{2}O\to H^{-}+OH$$

(3) $$OH\cdot +OH\cdot \to H_{2}O_{2}.$$

However, the H radical was not detected in both cases, which confirmed that water decomposition into the atomic H and OH∙ radical pathway was not dominant in our experiments. The presence of the N_2_ second positive system was caused by excitation, quenching processes, associative excitation, pooling reactions, transfer of energy between collisional partners, Penning excitation [26], etc. The N_2_ species were then combined into NO_3_^−^ and NO_2_^−^ long-lived species in the liquid. Although the energy of metastable-state Ar is lower than the threshold excitation energy of O_2_ (13.6 eV), metastable-state Ar can dissociate oxygen molecules and excite the state by Penning excitation. However, the O emission lines were not detected due to their low intensity.

**Figure 11:**
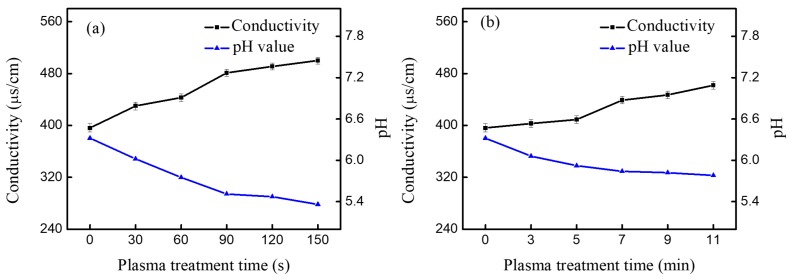
The conductivity and pH value of the mixture solution after plasma reaction: (**a**) A-Jet for 90 s (voltage: 6.4 kV, frequency: 90 kHz) and (**b**) P-Jet for 7 min (voltage: 8 kV, frequency: 8 kHz).

Furthermore, the liquid chemistry was studied by measuring the conductivity, pH value, and Cl^−^ concertation. The applied voltage for the A-Jet was kept at 6.4 kV, and it was 8 kV for the P-Jet in these experiments, unless otherwise specified. As shown in Figure 11a, the pH value decreased almost linearly with the increase of the plasma treatment time, while the conductivity showed the opposite tendency. After AC plasma treatment for 150 s, the pH value decreased from 6.32 to 5.36, while the conductivity increased from 396 to 500 μs/cm. With the pulsed power plasma treatment, the pH value and conductivity showed a tendency similar to the AC plasma jet, but with a smaller change. For example, after 11 min of treatment, the pH decreased from 6.32 to 5.78, and the conductivity increased from 396 to 462 μs/cm. The slower changes of pH and conductivity in the P-Jet compared with the A-Jet were consistent with its slower AuNP generation rate. A similar evolution of pH and conductivity was observed in discharges generated above the water surface [27]. The changes in pH and conductivity may be attributed to the water hydrolysis initiated by electrons or reactive species generated by the plasma jet [28]. Water hydrolysis produced H^+^ and led to a decreased pH value during the reaction:

(4) $$2H_{2}O-4e^{-}\to O_{2}↑+4H^{+}.$$

In addition, the reduction of Au^3+^ by H_2_O_2_ consumed OH^−^:

(5) $$3H_{2}O_{2}+3OH^{-}+Au^{3+}\to Au^{0}+3HO_{2}+3H_{2}O.$$

The pH variation in the solution was essential for AuNP diameter control. In the first state, the reduction of AuCl^−^ by plasma formed a loose self-assembly (100 nm or more) of small Au nanoclusters (less than 1 nm) [29]. With the pH drop in the solution, the self-assembly of Au nanoclusters disassembled into smaller sizes than the previous assembly. Because at low pH values, the self-assembly of Au nanoclusters are easy to disassemble. This explains why AuNP presented a much larger size when generated by P-Jet compared with the A-Jet. As confirmed by the optical emission spectra, newly generated NO_3_^−^, NO_2_^−^, and Cl^−^ long-lived species cascaded by N_2_ species might have been responsible for the conductivity increase in the liquid.

**Figure 12:**
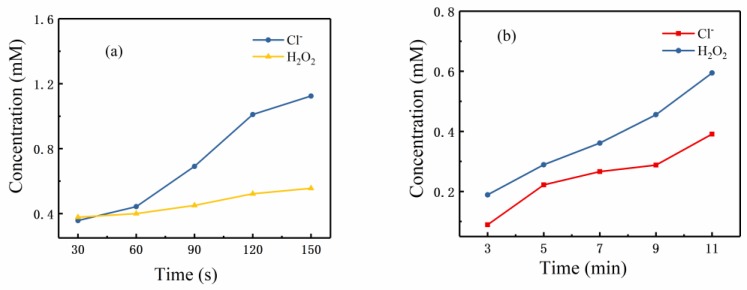
Cl^−^ and H_2_O_2_ generation in the (**a**) A-Jet and (**b**) P-Jet cases.

Figure 12 shows the Cl^−^ and H_2_O_2_ concentrations as treatment time increased for the A-Jet (a) and P-Jet (b). After plasma treatment, the H_2_O_2_ concentration was 0.56 mM for the A-Jet (reaction time: 150 s) and 0.59 mM for the P-Jet (reaction time: 11 min). Although the H_2_O_2_ concentration for the A-Jet and P-Jet processes reached almost the same value, clearly, the H_2_O_2_ production rate was much faster in the A-Jet process. The growth of Cl^−^ concentration was even faster in the A-Jet process. After a reaction time of 150 s, the Cl^−^ concentration reached 1.1 mM, while the Cl^−^ concentration was 0.4 mM in the P-Jet process after 11 min of reaction. These results proved that the strong chemical reactions involved in the A-Jet were responsible for its faster growth rate of AuNP.

### 3.4. The Role of Electrons and Neutral Species

**Figure 13:**
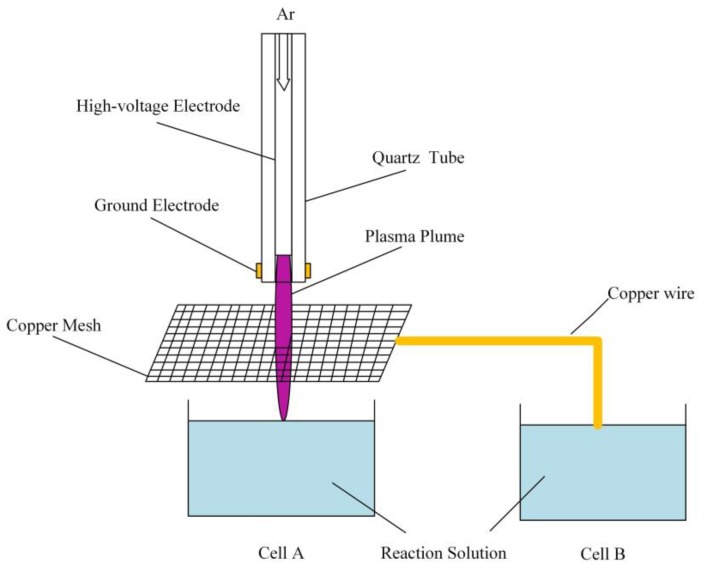
Schematic diagram of the experimental setup.

The generation of AuNP initiated the reduction of metal ions (Au^+^) in the solution either by electrons or neutral species. In the liquid cathode case [22], the Ar ions driven by the voltage fall attached to the liquid surface, creating some secondary effects, such as the generation of secondary electrons. The dissolved secondary electrons had a strong reducing ability for AuNP synthesis [30]. However, in our experiments, without a noble anode in the liquid, the voltage drop could be neglected, and the role of electron reduction could have been suppressed. To verify our assumption, a square copper grid with a diameter of 4 × 4 cm was placed above reaction cell A, and the copper grid was connected to a copper wire in cell B. So, only the electrons or ions could flux into cell B, while other reactive species were transported into cell A, and the effect of neutral species was studied. The schematic diagram of the experimental setup is shown in Figure 13. The validation of this experimental setup was proved in [31]. Cell C, which did not have a copper grid and wire, was studied as the control group.

**Figure 14:**
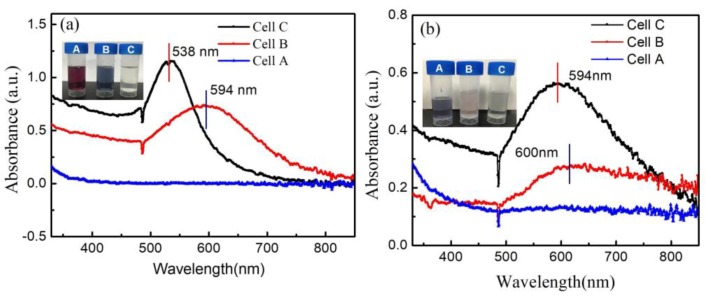
UV–Vis absorption spectra of AuNP for cells A–C with the interaction of (**a**) the A-Jet and (**b**) the P-Jet (A, B, and C in the vials stands for Cell A, Cell B and Cell C).

Figure 14 shows the UV–Vis spectra in these three cells after plasma treatment for different times (90 s for A-Jet and 7 min for P-Jet). Compared with cell C, cell B showed a red-shift absorption peak and a lower peak intensity. There were no absorption peaks detected in cell A, which confirmed that electron reduction alone was not dominant in our experiments. Visual observation indicated the same trend: the color of the AuNP changed from red-purple to dark-purple in the A-Jet case and changed from dark-purple to shallow pink. Cell A showed no color changes in both cases. These results also confirmed that the electrons and neutral species had a notable synergetic effect on AuNP synthesis, causing a higher absorption intensity in cell C.

**Figure 15:**
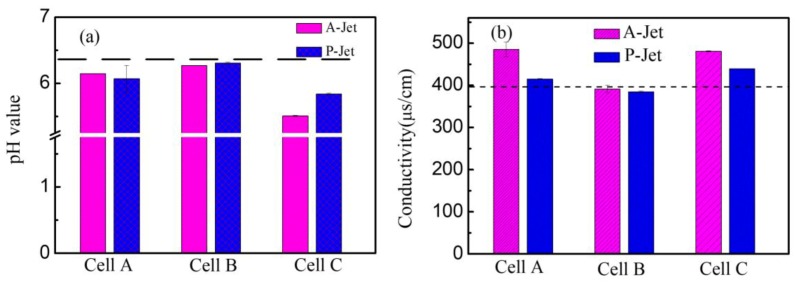
The conductivity and pH value of the different cells at: (**a**) A-Jet for 90 s (voltage: 6.4 kV, frequency: 90 kHz) and (**b**) P-Jet for 7 min (voltage: 8 kV, frequency: 8 kHz). The dashed line represents the initial pH value or conductivity of the mixture without plasma treatment.

As shown in Figure 15a, the variation of the pH value in Cell A, B, and C was 6.15, 6.27, and 5.51 for the A-Jet and 6.07, 6.31, and 5.84 for the P-Jet, respectively. This confirmed that the electrons transferred to the liquid had little effect on pH value changes. In cell A, the pH value reduced to 6.15 and 6.07 for the A-Jet and the P-Jet, respectively. The combination of natural species and electrons resulted in a maximum pH value reduction of 5.51 and 5.84 for the A-Jet and the P-Jet, respectively. Similar phenomena were observed for the conductivity measured in the three cells. An increase in conductivity was only observed in cells A and C. These results were consistent with the UV–Vis absorption spectra.

## 4. Discussions

### 4.1. Chemical Reactions for AuNP Generation

In the plasma–liquid interaction, chemical reactions typically occurred in the gas phase, the gas–liquid interface, and the liquid phase simultaneously. In the gas phase, plasma ignition generated high-energy electrons, reactive species, and UV. These species subsequently dissolved in the water and had a major role in influencing the final liquid chemistry [32]. As confirmed by optical emission spectroscopy (Figure 10), excited Ar, OH∙, and N_2_ (*C^3^Π_μ_-B^3^Π_g_*) were directly observed. Under a certain amount of pressure provided by Ar gas, there was a thin layer of water steam above the bulk liquid. The transportation of ions, electrons, and neutral species went through the plasma–liquid interface to the bulk liquid. In this region, the recombination, absorption, or desorption of reactive species and solvation of electrons or ions occurred [33], for example, the formation of hydrogen peroxide by hydroxyl radical recombination ($2OH_{\left(int\right)}\to H_{2}O_{2}{}_{\left(int\right)}$), followed by incorporation of the hydrogen peroxide into the liquid ($H_{2}O_{2}{}_{\left(int\right)}\to H_{2}O_{2}{}_{\left(aq\right)}$). For ions or low-energy electrons, they were mostly immediately solvated when striking the liquid. The simulations showed that even 100 eV O^+^ ions do not penetrate beyond the liquid surface by more than 3 nm [34]. As for high-energy electrons, the excitation, dissociation, or ionization of water molecules was expected. Solvated electrons (or hydrated electrons) can hydrolyze water by the following reactions:

(6) $$2e_{\left(\mathrm{aq}\right)}^{-}+2H_{2}O\to H_{2}{}_{\left(g\right)}+2OH^{-},$$

(7) $$2e_{\left(\mathrm{aq}\right)}^{-}+2H^{+}\to H+2OH^{-}.$$

Electrolytic reactions between plasma electrons and aqueous ions yield an excess of hydroxide ions (OH^−^), making the solution more basic, while reactions between reactive neutral species formed in the plasma phase and the solution lead to nitrous acid (HNO_2_), nitric acid (HNO_3_), and hydrogen peroxide (H_2_O_2_), making the solution more acidic [35]. According to our results, the pH value decreased rather than increased, confirming that the latter process dominated.

**Figure 16:**
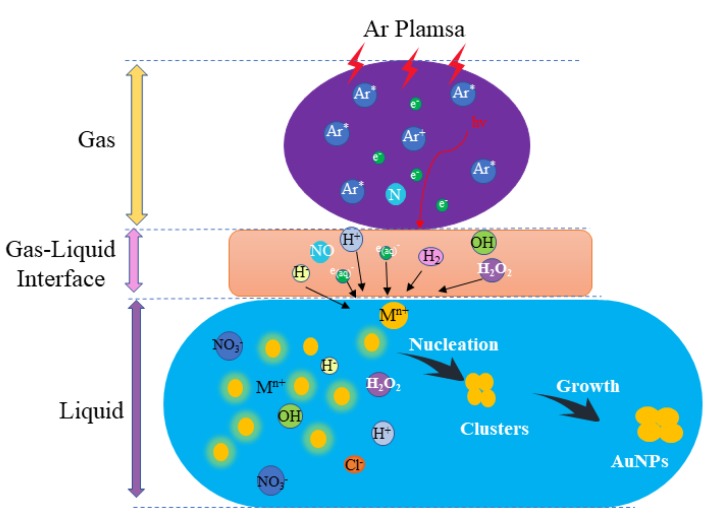
Schematic diagram of the synthesis mechanisms for plasma–liquid interaction.

In the bulk liquid, the reduction of AuCl^−^ by a reductant agent (H_2_O_2_, hydrated electrons) occurred. In general, reduction by both long-lived species (e.g., H_2_O_2_) and electrons was considered very important, but it is unclear if one was dominant. By putting a copper mesh over the reaction cell, the role of electrons and neutral species was studied. With electrons, there was no AuNP generation, while neutral species induced visible color changes with plasma treatment, confirming that the neutral species played a more important role for AuNP synthesis. It is interesting to note that there was a unique coupling effect between both reactions. This is because the generation of AuNP also requires a moderate pH value and conductivity. The complex processes of plasma–liquid chemistry for AuNP synthesis is shown in Figure 16.

### 4.2. The Effect of A-Jet and P-Jet

The breakdown mechanisms for the A-Jet and the P-Jet are different. For the A-Jet, filament discharge was observed, as confirmed by the waveform of the discharge current shown in Figure 2. Since the excitation voltage is a continuous sine AC wave voltage, the directional movement of ions cannot be neglected, which means the working gas can be heated by positive ions and neutral particles [36]. For the P-Jet, with the narrow pulse duration and short pulse rising time, the electrical energy was consumed to generate energetic electrons during discharge instead of heating plasma gas. The electron energy in P-Jet was higher than that of A-Jet. During one pulse discharge period, a short discharge duration time (about 200 ns, including positive and negative discharges) and a long discharge period (about 1.25 × 10^8^ ns) guaranteed that there was enough time for the plasma to cool down sufficiently. Thus, a prime difference between A-Jet and P-Jet was their different electron energy and gas temperature. As proved by many studies, the gas temperature of an A-Jet is always higher than that of a P-Jet. The temperature of a P-Jet can remain at room temperature, while an A-Jet can reach 100 °C in similar conditions [34]. During the reaction process, plasma directly touches with the liquid surface, and the reaction occurs mainly around this region. As is known, the reaction temperature is essential for AuNP synthesis, and a higher reaction temperature is beneficial for a higher reaction rate. So, the AuNP growth rate observed here was much higher in the A-Jet (0.4 mg/s) than in the P-Jet (9.5 μg/s). Due to the small diameter of the quartz tubing (the plasma was focused at a very small area of about 1.3 × 10^−2^ cm^−2^) and the short reaction time (especially for the A-Jet), the bulk liquid temperature has little changes. Although P-Jet favors a higher electron energy, as we confirmed in Section 3.4, the electron alone has a minor role in Au^+^ reduction.

Another consideration is the discharge power. With a short pulse rise time (50 ns), plasma is excited at a high overvoltage. In principle, more highly energetic electrons are produced in P-plasma. However, here, due to the short pulse duration time and small pulse frequency, the input power and energy of the P-Jet was only half that of the A-Jet. As we explained in Section 4.1, the electrons alone had little effect on Au^+^ reduction. Instead, the concentration of Cl^−^ and H_2_O_2_ were much higher in the A-Jet, and H_2_O_2_ can be the main reducing agent for Au^+^. The higher concentration of Cl^−^ was beneficial for the pH drop, which thus enhanced the dissociation of Au nanoclusters, resulting in a smaller AuNP diameter.

## 5. Conclusions

In this study, aqueous AuNP were successfully generated by A-Jet and P-Jet, respectively. Different AuNP synthesis processes were observed: (1) faster AuNP growth rate for the A-Jet (more than 40 times faster than the P-Jet) and (2) narrower AuNP size distribution and a broader size control range in the A-Jet case compared to P-Jet case. Further analysis revealed an increase in chemical concentrations (Cl^−^ and H_2_O_2_) and conductivity after plasma treatment and a higher increased amplitude in the A-Jet case. In addition, the pH value decreased during the reaction. The differences between the A-Jet and the P-Jet were due to their different discharge mechanisms: the local heating and higher discharge power in the A-Jet were very important for AuNP generation. Finally, by putting a copper mesh over the reaction cell, the roles of electrons and neutral species were studied. With electrons, there was no AuNP generation, while neutral species (e.g., OH∙) induced visible color changes with plasma treatment. The long-lived species (e.g., H_2_O_2_, combined by OH∙) was responsible for Au^3+^ reducing and played a more important role in AuNP synthesis.

## Figures and Tables

**Table 1:** Summarize of AuNP properties treated by A-Jet.

Parameters		A-Jet			
		Wavelength (nm)	Absorbance (a.u.)	Average Size (nm)	Particles Type
Time (s)	30	584	0.437	-	-
	60	566	0.772	-	-
	90	535	1.028	20.3 ± 12.2	Spherical, cylindrical, hexagon
	120	541	1.053	27.4 ± 10.4	Spherical, cylindrical, hexagon
	150	545	1.046	-	-
AuHCl_4_/Sodium citrate ratio	1:1	577	1.877	-	
	1:3	533	1.072	18.2 ± 9.0	Spherical, cylindrical, hexagon
	1:1.75	548	1.410	32.9 ± 14.1	Spherical, cylindrical, hexagon
	1:0.3	595	0.550	180.6 ± 20.5	Spherical, cylindrical, hexagon

**Table 2:** Summarize of AuNP properties treated by P-Jet.

Parameters		P-Jet			
		Wavelength (nm)	Absorbance (a.u.)	Average Size (nm)	Particles Type
Time (min)	3	590	0.977	-	-
	5	588	1.087	-	-
	7	582	1.167	81.2 ± 19.2	Spherical, cylindrical, hexagon
	9	587	0.779	100.2 ± 20.3	Spherical, cylindrical, hexagon
	11	586	0.694	95.3 ± 29.8	Spherical, cylindrical, hexagon
AuHCl_4_/Sodium citrate ratio	1:1	594	1.923	-	-
	1:3	582	1.167	82.5 ± 21.5	Spherical, cylindrical, hexagon
	1:1.75	589	1.285	-	-
	1:0.3	603	0.716	-	-
